# A dominance of Mu class glutathione transferases within the equine tapeworm *Anoplocephala perfoliata*

**DOI:** 10.1017/S0031182024000015

**Published:** 2024-03

**Authors:** Holly M. Northcote, Boontarikaan Wititkornkul, David J. Cutress, Nathan D. Allen, Peter M. Brophy, Ruth E. Wonfor, Russell M. Morphew

**Affiliations:** 1 Department of Life Sciences, Aberystwyth University, Aberystwyth SY23 3DA, UK;; 2 Faculty of Veterinary Science, Rajamangala University of Technology Srivijaya, Nakhon Si Thammarat 80240, Thailand

**Keywords:** *Anoplocephala perfoliata*, glutathione transferase, mu, omega, sigma, somatic

## Abstract

The most common equine tapeworm, *Anoplocephala perfoliata*, has often been neglected amongst molecular investigations and has been faced with limited treatment options. However, the recent release of a transcriptome dataset has now provided opportunities for in-depth analysis of *A. perfoliata* protein expression. Here, global, and sub-proteomic approaches were utilized to provide a comprehensive characterization of the *A. perfoliata* soluble glutathione transferases (GST) (ApGST). Utilizing both bioinformatics and gel-based proteomics, GeLC and 2D-SDS PAGE, the *A. perfoliata ‘*GST-ome’ was observed to be dominated with Mu class GST representatives. In addition, both Sigma and Omega class GSTs were identified, albeit to a lesser extent and absent from affinity chromatography approaches. Moreover, 51 ApGSTs were localized across somatic (47 GSTs), extracellular vesicles (EVs) (Whole: 1 GST, Surface: 2 GSTs) and EV depleted excretory secretory product (ESP) (9 GSTs) proteomes. In related helminths, GSTs have shown promise as novel anthelmintic or vaccine targets for improved helminth control. Thus, provides potential targets for understanding *A. perfoliata* novel infection mechanisms, host–parasite relationships and anthelmintic treatments.

## Graphical abstract

**Figure:**
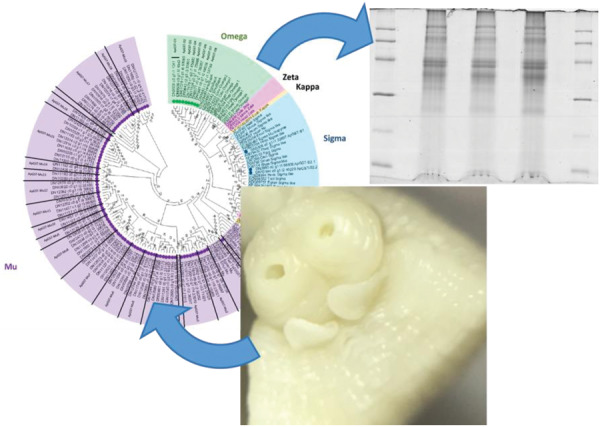

## Introduction

*Anoplocephala perfoliata* is recognized as the most common cestode to colonize the gastrointestinal tract of horses worldwide and is responsible for significant clinical disease (Gasser *et al*., 2005). At present, a dearth of molecular research on *A. perfoliata* restricts our understanding of the epidemiology and pathogenicity of infections and limits the potential to develop control tools such as vaccines and anthelmintics. For example, molecular diagnostic methods have demonstrated that these parasites are considerably more prevalent than previous coprological methods have suggested (Gasser *et al*., 2005; Lightbody *et al*., 2018). Furthermore, necropsy studies in areas of Europe and the US demonstrate prevalence has changed very little over the last 2 decades (Owen *et al*., 1988; Fogarty *et al*., 1994; Meana *et al*., 1998, 2005; Lyons *et al*., 2000, 2018; Lightbody *et al*., 2016). Finally, there are increased concerns for induced disease, such as colic, which can often be fatal (Gasser *et al*., 2005; Back *et al*., 2013). *A. perfoliata* has long been recognized as a serious equine pathogen (Proudman *et al*., 1998; Proudman and Trees, 1999). However, infection has also been regarded as unimportant among surveillance programs (Nielsen *et al*., 2006). Despite this, reports continue to demonstrate that infection poses significant risk to equine health. Indeed, intensifying burdens correlates with an increasing severity of inflammation, intestinal lesions, ulceration, thickening of the mucosa, submucosa, hypertrophy of circular muscle and colic (Williamson *et al*., 1997; Proudman *et al*., 1998; Proudman and Trees, 1999; Pavone *et al*., 2011; Lawson *et al*., 2019).

To date, treatment of *A. perfoliata* relies solely on anthelmintics, namely pyrantel (PRT) or praziquantel (PZQ), to which resistance has not yet been published. Importantly, improved diagnostics have paved the way for reducing the excessive use of anthelmintics (Lightbody *et al*., 2018). The discontinuation of the equine tapeworm wormer Equitape® in the U.K. means PZQ will only be available at equine outlets in combination with ivermectin or moxidectin used to treat Cyathostomins, potentially compromising future helminth control. However, PZQ is available as a single active under a ‘special’ consideration from veterinarians. Increasing use of combination anthelmintics as a control option could exacerbate existing resistance occurring within cyathostomins (Nielsen *et al*., 2020, 2022). These concerns for increased helminth anthelmintic resistance, in addition to the impact of *A. perfoliata* on equine health, make it imperative to maximize the efficiency of control options.

Given the importance of anthelmintic control of tapeworms, and wider helminth species, there has been increasing interest in using functional genomic approaches, such as proteomics, to understand how parasitic helminths metabolize anthelmintics. Of note has been the proteomic scrutinization of the glutathione transferase protein superfamilies (GST) from a number of helminths (Van Rossum *et al*., 2001; Chemale *et al*., 2006; Morphew *et al*., 2012). GSTs, as phase II detoxification enzymes, have a widespread distribution across parasite tissues, potentially accounting for up to 3% of soluble cytosolic proteins in cestodes (Brophy and Barrett, 1990*a*).

Helminth Sigma, Mu and Omega class GSTs are renowned for their importance in the host–parasite interaction, such as a major detoxification of xenobiotic compounds and endogenously derived toxins (Chemale *et al*., 2006; LaCourse *et al*., 2012; Morphew *et al*., 2012; Stuart *et al*., 2021). However, these detoxification mechanisms could also provide protection from oxidative membrane damage *via* immune assault, internal toxic compounds of helminth metabolism, as well as xenobiotics, such as anthelmintics (van Rossum *et al*., 2004; Kim *et al*., 2016). Members of the Omega class of GSTs are also suggested to major roles in the protection of the reproductive system during maturation and as an oxidative response (Board *et al*., 2000; Chemale *et al*., 2006; Burmeister *et al*., 2008; Morphew *et al*., 2012; Meng *et al*., 2014; Kim *et al*., 2016). Furthermore, helminth GSTs have further adapted to the host environment and demonstrate immunomodulatory activities to support establishment and duration (Sato and Kamiya, 1995; LaCourse *et al*., 2012; Wang *et al*., 2022). In particular, Sigma class GSTs are involved in prostaglandin synthase activity and a role in the modulation of the host immune response (Chemale *et al*., 2006; LaCourse *et al*., 2012). Consequently, research has shown evidence that helminth GSTs may have key roles in the establishment of infection and the development of anthelmintic resistance, as demonstrated in the parasitic flatworm *Fasciola hepatica* (Chemale *et al*., 2010; Scarcella *et al*., 2012). To this end, parasitic flatworm GSTs have shown potential as novel a vaccine candidate (Capron *et al*., 2005; Dowling *et al*., 2010; LaCourse *et al*., 2012).

Global and sub-proteomic studies on the somatic or extracellular proteomes of Anoplocephalidae are limited. However, the recent development of a transcriptome has provided an invaluable source of information for comprehensive molecular analysis of *A. perfoliata* (Wititkornkul *et al*., 2021). This development has led to the proteomic profiling of the *A. perfoliata* secretome including the free excretory/secretory proteins (ESP) and *A. perfoliata* derived extracellular vesicles (EVs) (Wititkornkul *et al*., 2021); demonstrating proteins released into the host milieu contain a variety of potential immune modulator molecules such as calpains, enolases and importantly GSTs. Thus, the foundation for utilizing in-depth proteomic approaches to better understand *A. perfoliata* at a molecular level has been provided. This allows the potential identification of the GST superfamily present in this parasitic cestode as has been previously employed for a number of helminths and protein superfamilies (Van Rossum *et al*., 2001; Morphew *et al*., 2012; Morphew *et al*., 2016). Here, global and sub-proteomic approaches were used to investigate the presence of GSTs derived from the equine cestode, *A. perfoliata*. In doing so, it provides the first evidence of functional expression of 3 *A. perfoliata* GST (ApGST) classes, namely Mu, Sigma and Omega class GSTs, at the protein level.

## Materials and methods

### Bioinformatic analysis of the *A. perfoliata* transcriptome

Potential GST protein sequences were initially identified within the *A. perfoliata* transcriptome (available at https://sequenceserver.ibers.aber.ac.uk/) according to (Wititkornkul *et al*., 2021). Briefly, known confirmed GST protein sequences were used in a tBLASTn (Altschul *et al*., 1997) against the in-house *A. perfoliata* transcriptome (Wititkornkul *et al*., 2021) through BioEdit Sequence Alignment Editor (Version 7.2.6.1; Hall, 1999) with the number of expected hits of similar quality (*e*-value) cut-off set at 1.0 × 10^−15^. Protein sequences of recognized GST superfamily classes included Alpha, Delta, Epsilon, Zeta, Theta, Mu, Nu, Pi, Sigma and Omega classes from mammals, insects and helminths; all of which were retrieved from Genbank and NCBI Reference Sequence (http://www.ncbi.nlm.nih.gov/) (Supplementary Table S1). Known sequences also included the confirmed Sigma class GSTs identified by Wititkornkul *et al*. (2021). The top hit sequences homologous to recognized GST sequences (lowest e-value) were taken as the representative *A. perfoliata* GST sequences, and subsequently translated into the protein sequences with ExPASy Translate tools (https://web.expasy.org/translate/; Gasteiger *et al*., 2003). Selected protein sequences were confirmed as a potential GST sequence by protein super-families, domain prediction and functional site analysis using InterProScan databases (version 77.0; http://www.ebi.ac.uk/interpro/; Jones *et al*., 2014; Blum *et al*., 2021) followed by searching against the NCBI (nr) protein database using a protein query (BLASTp; https://blast.ncbi.nlm.nih.gov/Blast.cgi; Altschul *et al*., 1997). The resulting InterPro domains classified as a Glutathione transferase superfamily with either C-terminal or N-terminal domain (IPR036282 and IPR004045 respectively) were kept as a unique GST protein sequence. Non-GST protein sequences, short protein sequences (less than 100 amino acids) and fragmented split sequences were excluded from future analysis. All unique classified GST sequences, or 1 representative if isoforms were present, were taken as a final *A. perfoliata* GST representative protein sequence for subsequent phylogenetic analysis.

### Multiple sequence alignment and phylogenetic analysis of *A. perfoliata* GSTs

The resulting *A. perfoliata* GST representative protein sequences were aligned with recognized GST protein sequences from 18 different species covering a range of mammals, nematodes, trematodes, cestodes and insects (Supplementary Table S1.) using ClustalW through BioEdit Multiple Sequence Alignment Editor (Version 7.2.6.1; Hall, 1999). Subsequently, an *A. perfoliata* GST phylogenetic tree was constructed and visualized in MEGA X (version 10.1.7; Hall, 2013; Kumar *et al*., 2018). Reliability of the phylogenetic tree was estimated with 1000 bootstrap replicates, using a Maximum Likelihood (ML) method.

### Bioinformatic analysis of Omega class GSTs

To investigate and confirm novel *A. perfoliata* Omega class GST sequences, potential *A. perfoliata* Omega class GST sequences clustering in previous phylogenetic analyses were aligned separately using ClustalW multiple sequence alignment and used for the generation of an Omega specific phylogenetic tree as described above. Subsequently, the characteristic secondary structure of potential *A. perfoliata* Omega class GST sequences was predicted using the Predict Secondary Structure (PSIPRED) Protein Analysis Workbench (PSIPRED 4.0; http://bioinf.cs.ucl.ac.uk/psipred/; Jones, 1999; Buchan and Jones, 2019). Omega class GST homologues were further investigated based on the glutathione (GSH)-binding site characteristic of Omega class GSTs in the N-terminal domain including the proline-rich residues in N-terminal extension (Morphew *et al*., 2012), the catalytic cysteine residues characteristic of Omega class GSTs (Morphew *et al*., 2012; Kim *et al*., 2016) and Omega class GST signature motifs (Chemale *et al*., 2006).

### Parasite collection

Live adult cestodes were manually extracted from the caecum at the ileocecal valve and the surrounding caecal walls of naturally infected horses immediately post-slaughter at a local commercial abattoir in Swindon, U.K. All obtained *A. perfoliata* were extensively washed in phosphate buffered saline to remove host material, flash-frozen, and stored at – 80°C until further use.

### Species verification

For DNA extraction, 20 mg of tissue was collected from 6 selected cestodes from the obtained individuals. DNA extraction was carried out using the DNeasy Blood & Tissue Kit (Qiagen Ltd) according to the manufacturer's instructions, as described previously (Morphew *et al*., 2012) and the concentration and purity of the extracted DNA quantified using NanoDrop (ThermoFisher Scientific, USA). Species verification PCR amplification was performed using ITS2 forward primer (5’-TGTGTCGATGAAGAGCGCAG) and ITS2 reverse primer (5’-TGGTTAGTTTCTTTTCCTCCGC), as described previously (Morphew *et al*., 2012). PCR products were subjected to 1% w/v agarose gel electrophoresis. PCR products were extracted from the gel using a Quick Gel Extraction and PCR Purification Combo Kit (Invitrogen, PureLink®) according to manufacturer's instructions and sequenced in-house using the primers described. Sequenced ITS2 regions were then identified by BLASTn against NCBI database.

### Cytosolic protein extraction

Replicates of 10 whole *A. perfoliata* were homogenized in a glass-glass homogenizer on ice in 20 mm potassium phosphate buffer (pH 7.4) containing 50 mm NaCl, 1% v/v Triton X-100 and a cocktail of protease inhibitors (Roche, Complete Mini, EDTA-free). Homogenized samples were delipidated through glass wool at 1000× g for 2 minutes. Samples were then centrifuged at 100 000× g for 45 minutes at 4°C and the soluble protein fraction collected. Protein concentration of the cytosolic protein was quantified using Bradford reagent (Sigma-Aldrich; Bradford, 1976).

### GST purification

GSTs were purified as described by Morphew *et al*. (2012). Briefly, the prepared cytosolic protein was filtered through 0.45 *μ*m syringe filter prior to being applied to a 1 mL Glutathione (GSH)-agarose affinity column (Sigma-Aldrich). The column was equilibrated with equilibration buffer (10 mm KPO4, 150 mm NaCl, pH 7.4) for 20 minutes at a flow rate of 1 mL/minute (20 × bed volumes). The cytosolic protein was then passed over the GSH-agarose 6 times to ensure maximal GST recovery and the flow through was collected. The column was washed with 20 × bed volumes of equilibration buffer to remove any non-specific bound proteins. GST proteins were eluted from the GSH-agarose using 5 mL of elution buffer (50 mm Tris-HCl, 7.5 mm GSH], pH 6.5). Eluted proteins were concentrated through 10 kDa ultra centrifugal filters (Amicon Ultra, Millipore) according to the manufacturer's instructions with a buffer exchange step to ddH_2_O. Purified GSTs and cytosolic protein flow through were quantified with Bradford Reagent (Bradford, 1976).

### GST functional assays

The CDNB (1-Chloro-2,4-dinitrobenzene) glutathione conjugation model substrate assay was used to measure GST activity (Habig *et al*., 1974). The assay was completed in a 1 mL volume, with a final concentration of 100 mm potassium phosphate buffer (pH 6.5), 1 mm reduced glutathione, 1 mm CDNB and absorbance change measured at 340 nm for 3 min at 20°C. A second model substrate, ethacrynic acid, was also tested according to the method previously described (LaCourse *et al*., 2012) using 1 mm GSH and 0.08 mm ethacrynic acid (EA) at pH 6.5.

### Protein precipitation

Whole cytosolic protein and purified GSTs were subjected to precipitation prior to electrophoresis. Briefly, a total of 100 *μ*g of somatic protein and 20 *μ*g of purified GSTs were precipitated with 20% v/v trichloroacetic acid (TCA) in ice cold acetone. After 1 h incubation at −20°C, samples were centrifuged at 21 000 × g to pellet proteins. Each protein pellet was then washed in ice cold 100% v/v acetone 3 times and air dried at −20°C for 15 mins. Precipitated cytosolic and GST samples were then resolubilized in isoelectric focusing buffer containing 8 M Urea, 2% w/v CHAPS, 33 mm DTT and 0.5% v/v ampholytes (pH 3–10).

### Gel electrophoresis

GST protein purification fractions or whole *A. perfoliata* cytosolic samples were run on 12.5% v/v 1D SDS-PAGE gels using the Protean® II xi 2-D Cell (Bio-Rad). Protein samples were mixed 1:1 with SDS loading buffer (0.2 M Tris-HCl, 8% SDS, 40% Glycerol, 0.02% Bronophenol blue, 50 mm DTT, pH 6.8), heated at 95°C for 5 mins and loaded. 1D gels were run at 70 V for 30 minutes followed by 150 V until completion. For 2D SDS-PAGE, GST samples were rehydrated and focused on 7 cm pH 3 − 10NL IPG strips to 10 000 Vh, using a Protean IEF Cell (Bio-Rad). Following focusing, IPG strips were equilibrated for 15 minutes in equilibration buffer (30% v/v glycerol, 6 M urea, 1% w/v DTT) followed by 15 min in alkylating equilibration buffer (30% v/v glycerol, 6 M urea, 4% w/v iodoacetamide). Equilibrated strips were run on 12.5% SDS-PAGE gels at 70 V for 30 minutes and 150 V until completion with Protean® II xi 2-D Cell (Bio-Rad). All gels were fixed for 1 hour in 10% v/v acetic acid and 40% v/v ethanol followed by Colloidal Coomassie blue staining (Phastgel Blue R, Amersham Biosciences) overnight. Gels were de-stained in 1% v/v acetic acid and imaged using a GS-800 calibrated densitometer (BioRad). Individual GST protein spots observed on 2D SDS PAGE were detected and quantified using Progenesis PG220, software version 2006 (Nonlinear Dynamics Ltd.), as described previously (Morphew *et al*., 2014;, 2016).

### GST classification via immunoblotting

*A. perfoliata* cytosolic protein and purified GSTs were run on 1D or 2D SDS PAGE gels. Proteins were then transferred to a nitrocellulose membrane over 2 hours at 40 V according to the method of Towbin *et al*. (1979). Post transfer, membranes were stained with amido black (0.1% w/v Amido black, 10% v/v acetic acid and 25% v/v isopropanol) to monitor protein transfer, then de-stained 10% v/v acetic acid and 25% v/v isopropanol. De-stained membranes were washed with Tris buffered saline with Tween-20 (TTBS: 20 mm Tris-HCl,154 mm NaCl, 1% v/v Tween 20, pH 7.5) 3 times for 5 minutes. Membranes were then incubated overnight in blocking buffer (5% w/v skimmed milk powder in TTBS) prior to incubating membranes with primary antibodies. Primary antibodies, at 1:5,000 dilution for anti-Mu class antibody (SjGST26; Pharmacia-Biotech 27-4577) and 1: 30 000 for anti-Sigma class GST antibody (FhGST-S1; LaCourse *et al*., 2012), were incubated with the membrane at room temperature for 1.5 hours. Membranes were then washed for 5 minutes in Tris buffered saline (TBS; 20 mm Tris-HCl, 154 mm NaCl, pH 7.5) 3 times. Anti-Mu and anti-Sigma probed membranes were incubated in a secondary antibody diluted at 1: 30 000 in TTBS (anti-Rabbit IgG, conjugated with alkaline phosphatase, produced in Goat, Sigma-Aldrich) and immunogenic proteins visualized using BCIP/NBT (5-bromo-4-chloro-3-indoyl phosphate/nitro blue tetrazolium) liquid substrate system (Sigma-Aldrich). Membranes were images using the GS-800 calibrated densitometer (BioRad).

### Trypsin digestion and mass spectrometry

*A. perfoliata* somatic protein resolved on 1DE gels were subjected to GeLC proteomics. Each lane was cut into a series of 10 ‘bands’. Protein spots resolved from the purified GST were manually excised from the 2DE gels and used for classical in gel digestion. Protein bands and spots excised from the 1DE and 2DE gels were subjected to trypsin digestion prior to tandem mass spectrometry.

Gel pieces were de-stained and digested overnight in 10 ng/*μ*L trypsin, as previously described (Rooney *et al*., 2022). Immediately prior to performing tandem mass spectrometry the dried peptides were re-suspended in 20 *μ*L 0.1% v/v formic acid. For 2D SDS PAGE protein spots, the method of Duncan *et al*. (2018) was utilized for LC-MSMS. For whole *A. perfoliata* Orbitrap Fusion™ Tribrid™ mass spectrometer (Thermo Scientific™), with EASY-Spray™ Source, coupled to an UltiMate™ 3000 RSLCnano System (Thermo Scientific™) as per methods of Rooney *et al*. (2022).

### Protein identification and GST localization

Mass Hunter Qualitative Analysis software (V B.06, Agilent Technologies) was used to generate peak lists. Peak lists were then exported as MASCOT Generic Files. Whole worm and purified GST samples were submitted to an MSMS ion search using MASCOT against the recently produced *A. perfoliata* transcript database (Wititkornkul *et al*., 2021). Parameters for the MASCOT search were set as the following conditions: fixed modification of carbamidomethylation, variable modification set for oxidation of methionines, peptide charge to 2+, 3+ and 4+, instruments used ESI-QUAD-TOF (purified GSTs fractions) and ESI-TRAP (Whole somatic proteome). Identified peptides presenting MASCOT ions scores above 44 for whole cytosolic proteins and 45 for purified GSTs were considered to have a significant identity or extensive homology (*p* < 0.05).

## Results

### Characterization of novel *A. perfoliata* GSTs

The initial identification of *A. perfoliata* GST sequences within an in-house *A. perfoliata* transcriptome by tBLASTn produced a total of 330 *A. perfoliata* putative GST sequences based on homology to recognized GST superfamily sequences from mammals, insects and helminths (cutoff 1 × 10–15). Of these potential *A. perfoliata* GST sequences, each were classified using InterProScan and BLASTp, leading to 309 sequences predicted as GST superfamily membership (IPR040079), of which 59 sequences were initially predicted as Glutathione transferase, Mu class membership (IPR003081) (Supplementary Table S2).

**Figure 1.**
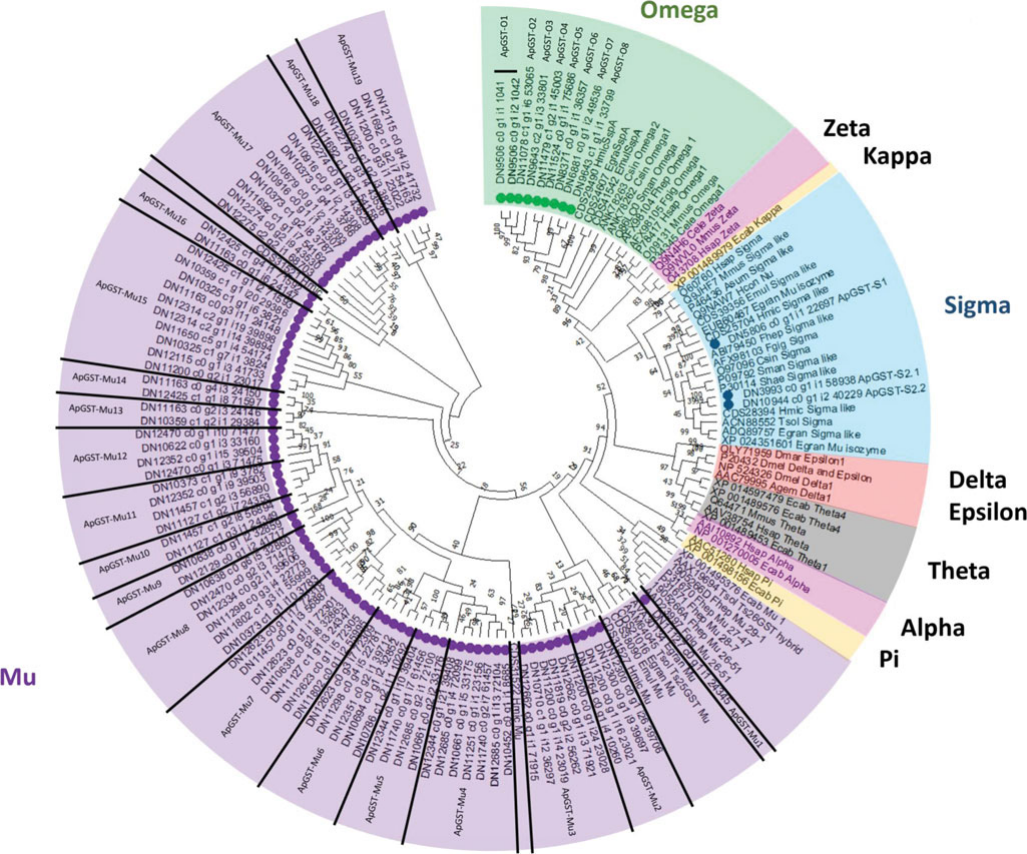
Phylogenetic analysis of Glutathione transferases (GSTs) identified within the *A. perfoliata* transcriptome inferred using a Maximum likelihood (ML) tree with JTT matrix-based model. The bootstrap consensus tree is inferred from 1000 replicates. Evolutionary analyses were conducted in MEGA X. Circles denote sequences identified within the bioinformatics analysis of *A. perfoliata*.

Prior to phylogenetic tree construction, several sequences were filtered out where isoforms were presented (180), short sequences (19) and alternative splice variants or fragmented sequences (15). A maximum likelihood (ML) phylogenetic analysis of the remaining 95 *A. perfoliata* potential GST representative sequences (Supplementary Table S2) and 58 recognized GST sequences demonstrated that there were no *A. perfoliata* GST sequences belonging to the Zeta, Delta, Epsilon, Theta, Alpha, Kappa and Pi classes (Fig. 1). Among the group of GST classes, the largest cluster of 83 *A. perfoliata* GST sequences were clustered in a Mu class GST group, while 3 and 9 *A. perfoliata* GST sequences were clustered, with strong bootstrap support, in the Sigma (64%) and Omega (98%) GST class groups, respectively (Fig. 1). Of note, only the 3 Sigma class representatives identified previously were identified within the Sigma class.

In total the 83 sequences that clustered into 1 large Mu class GST group, alongside known Mu class GSTs, broadly demonstrated 19 clades of *A. perfoliata* Mu class GSTs. All clades contained more than 1 ApGST-Mu representative, except for ApGST-Mu1 which contained a solitary member. ApGST-Mu1 was the member most closely linked to known platyhelminth Mu class GSTs including members from Fasciolids and *Taenia solium*, as well as the inclusion of a host, equid, Mu class GST (Fig. 1). Of interest were ApGST-Mu class 16 and 17 which formed a clade with a Mu class representative from *Hymenolepis microstoma* (Fig. 1).

### Characterization of novel *A. perfoliata* Omega class GSTs

**Figure 2.**
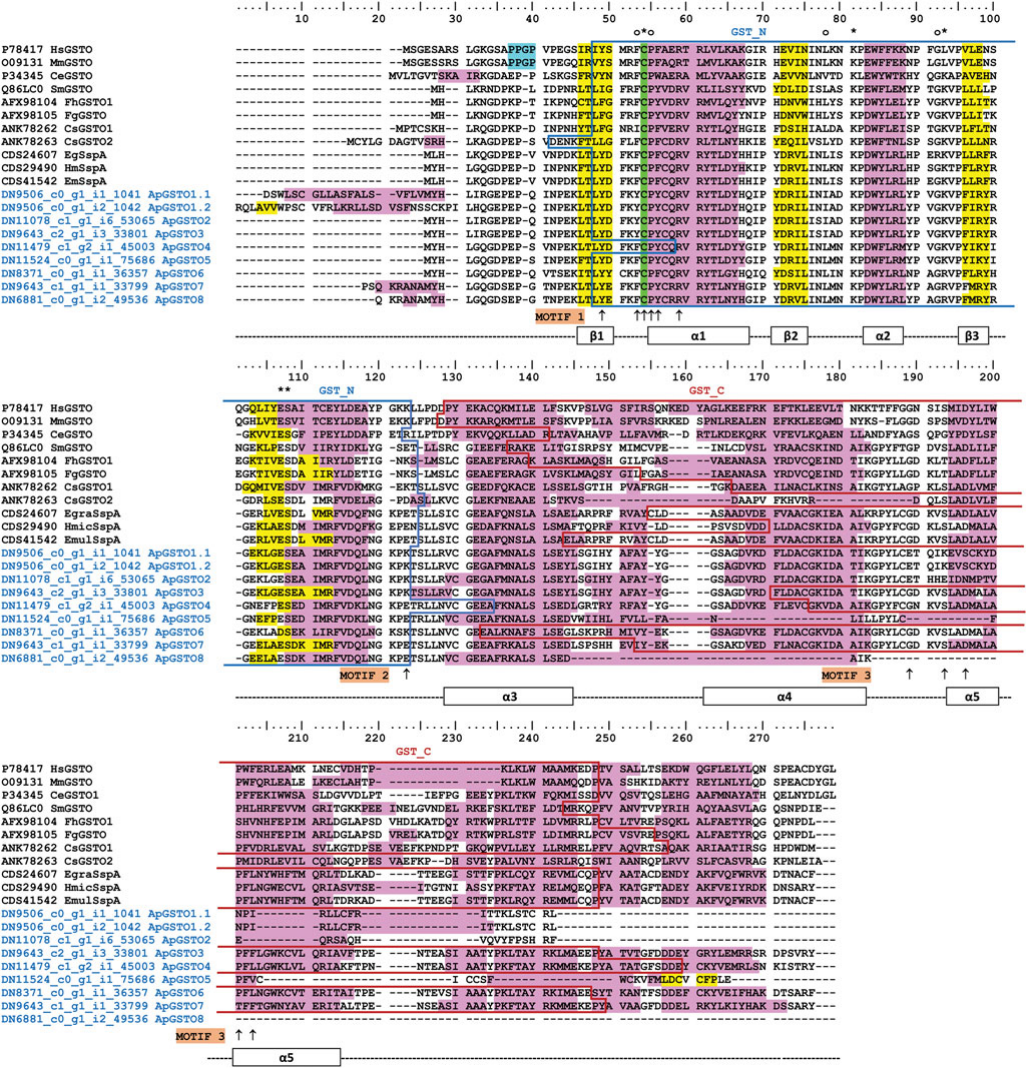
Multiple sequence alignment of the 8 novel Omega class GSTs identified in *A. perfoliata* (ApGST-O1.1 to ApGST-O8). Secondary protein structure prediction using the PSIPRED Protein Analysis Workbench is presented below the alignment is comprised of 3 *β*-strands, shaded in yellow and 5 *α*-helices, shaded in pink. The proline-rich residues in the Omega class characteristic N-terminal extension as denoted by Morphew *et al*. (2012) are shaded in blue. The catalytic cysteine residues characteristic of Omega class GSTs as denoted by Morphew *et al*. (2012), Meng *et al*. (2014) and Kim *et al*. (2016) are shaded in green. Amino acids residues directly contact to glutathione are indicated by asterisks (*) (Burmeister *et al*., 2008; Kim *et al*., 2016). Glutathione-binding residues are indicated by closed circles (o) (Kim *et al*., 2016). The Omega class GST signature motifs as denoted by Chemale *et al*., (Chemale *et al*., 2006) are marked by arrows (↑). The predicted N- and C-terminal GST domain profiles are indicated by blue- and red-boxes.

A total of 8 novel *A. perfoliata* Omega class GSTs represented by 9 sequences used within the phylogenetics (designated ApGST-O1 [1.1 and 1.2], ApGST-O2, ApGST-O3, ApGST-O4, ApGST-O5, ApGST-O6, ApGST-O7 and ApGST-O8) clustered within the Omega class GST branch within the whole GST phylogenetic analysis. All 9 sequences were further subjected to a multiple sequence alignment with 11 recognized Omega class GST sequences from mammals and helminths separately from the remaining GSTs (Fig. 2). The secondary characteristic structure of the multiple aligned *A. perfoliata* Omega GST homologue sequences predicted 3 *β*-strands and 5 *α*-helices demonstrating the consistency of the secondary characteristic structure between ApGST-O1.1 to ApGST-O8 and the 11 recognized Omega class GST sequences (Fig. 2). The investigation of Omega class GST sequences based on the GSH-binding sites in the N-terminal domain demonstrated that all 8 novel *A. perfoliata* Omega class GST sequences demonstrated a high homology to other GSH-binding sites (Burmeister *et al*., 2008; Kim *et al*., 2016), with the conserved catalytic cysteine residue characteristic of Omega class GSTs particularly at amino acid position 32 (Cys32) (Morphew *et al*., 2012; Meng *et al*., 2014; Kim *et al*., 2016). However, each lacked the proline-rich residues in the Omega class characteristic N-terminal extension (PXXP motif) (Morphew *et al*., 2012). Additionally, all 8 *A. perfoliata* Omega GSTs were confirmed as Omega class GST according to the presence of amino acid residues related to the Omega class GST signature motifs identified by Chemale *et al*. (2006) (Fig. 2). Of note, the sequence length of ApGST-O8 was significantly truncated with much of the C-terminal region absent. Therefore, ApGST-O8 was excluded from further phylogenetic analysis.

A maximum likelihood (ML) phylogenetic tree generated from 7 of the 8 novel *A. perfoliata* Omega class GST sequences (8 sequences; ApGST-O1.1 to ApGST-O7) and 11 recognized Omega class GST sequences demonstrated that all 7 *A. perfoliata* Omega class GST sequences clustered strongly together with good support (Bootstrap value 90%). In addition, the ApGST Omega class representatives were clustered, again with strong support (96%), with the homologous Stringent starvation protein A (SspA) sequences from the Cestode clade including representatives from *H. microstoma* (CDS29490), *Echinococcus granulosus* (CDS24607) and *E. multilocularis* (CDS41542) (Supplementary Figure S1).

### Species confirmation and GST activity

Live adult cestodes were obtained from the ileocecal valve from naturally infected horses and utilizing DNA extraction and PCR amplification of the ITS2 region were confirmed as *A. perfoliata* (Supplementary Figure S2). Following confirmation, homogenization of replicates of 10 worms yielded an average of 51.28 mg of cytosolic protein with a total GST activity of 490.34 *μ*mol min^−1^ and a GST specific activity of 9.56 ± 4.02 *μ*mol min^−1^ mg^−1^ prior to GSH based purification (Table 1.). Post GSH affinity chromatography, 0.33% of the total protein purified as GSTs with a specific activity recorded at 32.47 *μ*mol min^−1^ mg^−1^ (Table 1). However, given a loss of total activity post purification it is likely that the contribution of GSTs to the total proteome is much higher than 0.33%. In addition, purified ApGST demonstrated significant activity towards EA with a specific activity of 1069.8 ± 194.7 nmol min^−1^ mg^−1^.

**Table 1.** *A. perfoliata* GST activity of the cytosolic protein sample pre and post purification against the model substrate CDNB in relation to the total protein used from the sample.

	Total protein (mg)		Total GST activity (*μ*mol min^−1^)		Specific GST activity (mean ± s.d.) (*μ*mol min^−1^ mg^−1^)	
Sample	Pre	Post	Pre	Post	Pre	Post
Protein	51.28	0.17	490.24	5.52	9.56 ± 4.02	32.47 ± 3.06

Average taken across the 3 replicates.

### Immunological classification

**Figure 3.**
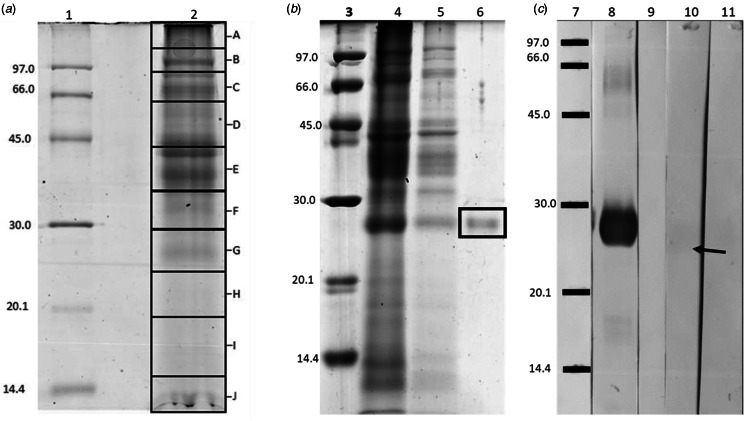
Representative 1DE SDS PAGE analysis of the purified *A. perfoliata* GSTs and western blotting. (A) 1DE of the whole cytosolic protein fraction – (1) Low molecular weight markers (GE Healthcare) – kDa and (2) 10 *μ*g of cytosolic protein fraction extracted from adult *A. perfoliata* (B) 1DE of the GSH affinity purification fractions demonstrating GST purification – (3) Low molecular weight markers (GE Healthcare) – kDa (4) 15 *μ*l of flow through after GSH affinity, (5) 20 *μ*l of GSH affinity wash and (6) 2 *μ*g of purified *A. perfoliata* GSTs (boxed). (C) Western blot of the purified *A. perfoliata* GSTs run on 1DE –and probed with either Anti-Sigma class or Anti-Mu class antibodies (7) Low molecular weight markers (GE Healthcare) – kDa, (8) *A. perfoliata* GSTs probed with Anti-Mu class antibodies, (9) Anti-Mu class negative control, (10) *A. perfoliata* GSTs probed with Anti-Sigma class antibodies, anti-FhGST-S1 and (11) Anti-Sigma class negative control. Lanes 8–11 all contained 2 *μ*g of ApGSTs. Very faint reaction to Sigma class GSTs is indicated by an ←. All 1D gels were run on 12.5% acrylamide.

For initial classification of purified ApGST, cytosolic proteins which had been purified through GSH-agarose affinity column and profiled using 1DE were transferred to nitrocellulose membranes and subjected to immunoblotting with parasitic flatworm Anti-Mu and Anti-Sigma class GST antibodies. For Mu class GSTs, membranes probed with the anti-Mu class antibody demonstrated a strong recognition from the 1DE band of purified ApGST indicating a potential abundance of Mu class representatives (Fig. 3). Regarding Sigma class GSTs, transferred 1DE profiles were probed with anti-FhGST-S1 antibodies (Fig. 3) and, in contrast, demonstrated faint reactivity suggesting a lack of purified Sigma class representatives.

### Proteomics of ApGSTs

Three biological replicates of whole *A. perfoliata* cytosolic proteins were resolved on 1D SDS-PAGE gels (Fig. 3) and identified through MASCOT *via* MS/MS Ion Search against the *A. perfoliata* transcriptome. ApGST representatives identified in the *A. perfoliata* transcriptome were then used to mine the cytosolic protein dataset, along with the *A. perfoliata* secretome (EV, EV surface and EV free ESP protein data) generated previously by Wititkornkul *et al*. (2021), for GSTs. In total, 51 unique ApGST sequences were observed across the 4 datasets (Table 2). The whole worm proteome produced the most abundant number of GST hits with 47 unique ApGST sequences identified within the 797 total proteins identified (Table 2, Supplementary Table S3). Two of which, ApGST-Mu11 (DN11457_c1_g2_i3_56890) and ApGST-Mu9 (DN12300_c0_g1_i9_39697), were present in the top 50 most abundant proteins. Additionally, within the *A. perfoliata* secretome 1, 2 and 9 GST proteins were observed for EVs, the EV surface and EV-depleted ESP protein datasets, respectively. Only 1 GST protein, ApGST-Mu3 (DN12662_c0_g1_i1_71915), was consistent across all 4 datasets with 6 GST representatives consistent across at least 2 fractions.

**Table 2.** ApGST proteins observed from mining of the *A. perfoliata* proteomic datasets from the whole worm, the EV, the EV surface and the EV-Depleted ESP

Accession	MASCOT score	Predicted GST class	Peptides matches	Unique peptides	Rank
**Whole cytosolic proteome**					
DN11457_c1_g2_i3_56890	394	ApGST-Mu11^T^	67	17	38
DN12300_c0_g1_i9_39697	286	ApGST-Mu9^T^	60	16	48
DN12662_c0_g1_i1_71915	301	ApGST-Mu3^T^	51	12	67
DN12334_c0_g2_i1_39606	181	ApGST-Mu8^T^	50	14	70
DN12470_c0_g1_i10_71477	239	ApGST-Mu12^T^	49	16	74
DN10764_c0_g1_i15_10263	263	Mu^H^	46	12	82
DN10373_c1_g1_i11_3785	282	Mu^H^	38	13	118
DN10786_c1_g2_i11_10292	285	ApGST-Mu6^T^	33	15	139
DN11163_c0_g1_i4_24144	153	Mu^H^	32	9	154
DN10764_c0_g1_i4_10260	503	ApGST-Mu2^T^	32	9	156
DN11692_c1_g1_i7_54162	113	ApGST-Mu17^T^	30	12	172
DN10325_c1_g7_i1_3824	237	ApGST-Mu15^T^	29	10	179
DN10710_c1_g1_i18_36299	229	Mu^H^	28	9	194
DN11298_c0_g4_i5_22781	168	ApGST-Mu6^T^	26	9	207
DN10373_c1_g1_i10_3783	166	ApGST-Mu8^T^	26	9	209
DN11200_c0_g1_i15_23020	236	Mu^H^	26	9	211
DN10661_c0_g1_i5_33175	97	ApGST-Mu4^T^	25	9	215
DN12300_c0_g1_i17_39701	195	Mu^H^	24	4	223
DN10373_c1_g2_i11_3788	72	Mu^H^	23	7	233
DN11802_c0_g1_i2_55995	204	ApGST-Mu7^T^	22	13	245
DN12300_c0_g1_i32_39710	135	Mu^H^	21	8	261
DN12623_c0_g3_i3_72304	76	Mu^H^	21	10	263
DN11200_c0_g3_i1_23022	140	ApGST-Mu19^T^	21	11	268
DN11163_c0_g3_i8_24143	217	Mu^H^	20	5	271
DN11479_c1_g2_i1_45003	281	ApGST-O4^T^	19	11	286
DN11200_c0_g1_i22_23025	389	Mu^H^	18	5	297
DN12274_c0_g2_i11_43533	79	Mu^H^	18	5	307
DN10373_c1_g1_i9_3782	97	ApGST-Mu11^T^	15	10	315
DN11692_c1_g2_i2_54157	197	Mu^H^	15	9	363
DN10671_c2_g1_i9_7021	133	Mu^H^	15	9	364
DN11127_c1_g1_i3_24347	277	Mu^H^	15	9	373
DN10452_c0_g1_i1_8685	107	ApGST-Mu4^T^	14	8	375
DN12662_c0_g1_i13_71921	131	ApGST-Mu3^T^	12	9	403
DN11740_c0_g1_i10_61458	123	Mu^H^	12	6	450
DN12351_c0_g2_i2_39713	135	Mu^H^	11	4	459
DN10764_c0_g1_i12_10262	97	Mu^H^	11	7	475
DN5806_c0_g1_i1_22697	111	ApGST-S1^T^	11	7	476
DN10786_c1_g1_i7_10293	79	Mu^H^	11	8	477
DN10325_c1_g6_i3_3823	144	Mu^H^	9	7	484
DN11650_c5_g1_i13_54177	127	Mu^H^	9	4	521
DN11298_c0_g3_i8_22780	49	Mu^H^	8	5	545
DN10359_c1_g1_i23_29387	122	Mu^H^	7	4	577
DN10661_c0_g2_i2_33176	83	ApGST-Mu5^T^	7	5	586
DN11802_c0_g1_i1_55994	62	Mu^H^	7	6	619
DN9643_c2_g1_i3_33801	64	ApGST-O3^T^	6	6	624
DN12662_c0_g1_i10_71920	78	Mu^H^	6	5	646
DN11251_c1_g1_i1_23160	72	Mu^H^	1	1	792
**EV proteome**					
DN12662_c0_g1_i1_71915	272	ApGST-Mu3^T^	13	10	71
**EV Surface proteome**					
DN12662_c0_g1_i1_71915	242	ApGST-Mu3^T^	11	8	139
DN12623_c0_g3_i3_72304	76	Mu^H^	2	2	291
**EV-Depleted ESP proteome**					
DN12662_c0_g1_i13_71921	585	ApGST-Mu3^T^	41	22	131
DN11479_c1_g2_i1_45003	343	ApGST-O4	17	11	145
DN11127_c0_g1_i1_24345	56	ApGST-Mu1^T^	12	2	200
DN10764_c0_g1_i12_10262	309	Mu^H^	14	9	249
DN11457_c1_g2_i3_56890	149	ApGST-Mu11^T^	15	3	321
DN10764_c0_g1_i19_10264	113	Mu^H^	9	4	334
DN12334_c0_g3_i2_39609	132	Mu^H^	7	3	350
DN5806_c0_g1_i1_22697	70	ApGST-S1	12	3	352
DN12662_c0_g1_i1_71915	125	ApGST-Mu3^T^	10	6	419

EV, EV surface and EV-Depleted ESP datasets obtained from Wititkornkul *et al*. (2021)

Dark grey highlights indicate GSTs present in all 4 proteomes.

Light grey highlights are those present across 2 proteomes.

T GST Class predicted by phylogenetic analysis

H Class predicted by homology to isoforms.

Of note, a dominance of Mu class GST members was observed across the complete datasets. In total, 44 out of the 47 unique ApGST sequences identified in the whole worm proteome were classified as, or homologous to, Mu class GST representatives (Table 2). Similarly, all of the ApGST sequences observed in the EVs and EV surface, and 7 of the 9 ApGST sequences in EV-depleted ESP were also classified, or homologous to, Mu class GST representatives. Regarding Sigma class GSTs, ApGST-S1 has previously been highlighted as the only Sigma class GST in the tapeworms secretome (Wititkornkul *et al*., 2021). Here, ApGST-S1 was also observed as the only Sigma class GST representative to be localized within the whole worm proteome with a similar relative low abundance. Finally, 2 of the novel *A. perfoliata* Omega GST representatives were identified in both the whole worm and EV-depleted ESP proteome. Specifically, ApGST-O3 was observed in both the whole worm protein and EV-depleted ESP proteins, whereas ApGST-O4 was only present in the EV-depleted ESP proteins.

### Proteomic profiling of affinity purified ApGSTs

**Figure 4.**
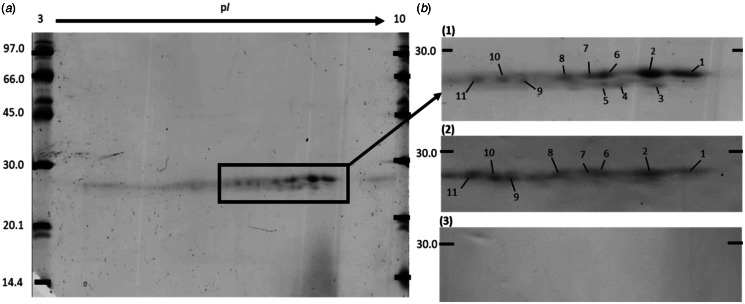
Representative 2DE SDS PAGE analysis of the purified *A. perfoliata* GSTs and western blotting. (A) 2DE SDS PAGE analysis of the purified *A. perfoliata* GSTs (20 *μ*g). (B) 2DE SDS PAGE analysis of the purified *A. perfoliata* GSTs (20 *μ*g) and subsequent western blotting – (1) Resolved spots of 2DE of GSH affinity purification fractions colloidal Coomassie blue stained. Numbered spots correspond to the putative protein identifications in Table 3. (2) Western blot of the purified *A. perfoliata* GSTs run on 2DE and probed with anti-Mu class antibodies, protein spots with prominent visible reactions are labelled corresponding to 2DE gel. (3) Western blot of the purified *A. perfoliata* GSTs run on 2DE and probed with anti-Sigma class, anti-FhGST-S1, antibodies. Position of low molecular weight marker (GE Healthcare) indicated – kDa. GSH affinity purification fractions were isoelectric focused on 7 cm pH 3 − 10 nonlinear IPG strips and run on 12.5% polyacrylamide gels. Gels for immunoblotting were transferred onto nitrocellulose membrane membranes for antibody binding and developed using the BCIP/NBT system.

GSH agarose affinity purified ApGSTs were resolved on 2D SDS-PAGE gels (Fig. 4). The 2D gels were then subjected to either MASCOT *via* MS/MS Ion Search against the *A. perfoliata* transcriptome or transferred to nitrocellulose membranes and subjected to immunoblotting with Mu and Sigma GST antibodies. The 2DE profiling of the purified ApGST fraction using GSH-agarose affinity column presented a total of 11 resolved protein spots ranging from 4.36 to 5.69 in pI and 23.93 to 24.86 kDa in size (Fig. 4., Table 3). Six of these protein spots were consistent across all biological replicates with the remaining 5 consistent across 2 replicates. The visible protein spots (1–11, Fig. 4) present on the replicate 2DE profiles were excised for MSMS analysis. All protein spots contained proteins with significant identity or extensive homology to ApGST representatives (Table 3; Supplementary Table S4). Three spots (Spots 4, 7 and 8) contained 1 additional protein that were not identified as ApGSTs (Importin subunit beta 1 [DN12350_c0_g1_i1_64773], General vesicular transport factor p115 [DN11790_c1_g5_i1_61055] and a Tetratricopeptide repeat protein 35 B [DN8984_c0_g1_i1_28059], respectively). The unique ApGST sequences hit from the 2D spots were solely classified as, or homologous to, Mu class GST representatives (Table 3).

**Table 3.** GST proteins identified in the whole worm cytosolic fraction of *A. perfoliata* following GSH agarose affinity chromatography. The top member of each protein family identified *via* MASCOT search is reported, remaining family members of significance are shown in Supplementary Table S4.

Spot	Normalized spot volume	Accession	MASCOT score	Predicted GST class	Peptides matches	Unique peptides	Sequence coverage (%)
1	23635	DN12662_c0_g1_i1_71915	953	ApGST-Mu3^T^	93	13	52.3
2	3824	DN12662_c0_g1_i1_71915	1000	ApGST-Mu3^T^	113	13	52.3
3	4559	DN12662_c0_g1_i1_71915	215	ApGST-Mu3^T^	20	7	33.9
4	8752	DN10373_c1_g1_i9_3782	211	ApGST-Mu11^T^	32	9	27.6
		DN12662_c0_g1_i1_71915	178	ApGST-Mu3^T^	13	5	20.9
		DN10786_c1_g2_i11_10292	88	ApGST-Mu6^T^	17	5	21.2
		DN12350_c0_g1_i1_64773	49	Importin subunit beta 1	4	3	3.2
5	9744	DN12662_c0_g1_i1_71915	179	ApGST-Mu3^T^	19	6	26.4
		DN10373_c1_g1_i9_3782	95	ApGST-Mu11^T^	32	9	29.4
		DN10786_c1_g2_i7_10291	66	Mu^H^	10	4	14.2
		DN11200_c0_g1_i21_23024	53	Mu^H^	6	3	26.5
6	4049	DN12662_c0_g1_i1_71915	734	ApGST-Mu3^T^	72	11	52
7	6582	DN12662_c0_g1_i1_71915	495	ApGST-Mu3^T^	53	11	52
		DN12350_c0_g1_i1_64773	56	Importin subunit beta 1	3	1	1
		DN11790_c1_g5_i1_61055	50	General vesicular transport factor p115	1	1	1.6
8	6760	DN12662_c0_g1_i1_71915	411	ApGST-Mu3^T^	47	11	52
		DN8984_c0_g1_i1_28059	52	Tetratricopeptide repeat protein 35 B	4	1	3.2
9	6219	DN12662_c0_g1_i1_71915	634	ApGST-Mu3^T^	70	11	52
10	8340	DN12662_c0_g1_i1_71915	406	ApGST-Mu3^T^	49	11	52
11	15143	DN12662_c0_g1_i1_71915	259	ApGST-Mu3^T^	31	8	36.5
		DN10373_c1_g1_i9_3782	81	ApGST-Mu11^T^	16	7	21.8
		DN10661_c0_g1_i5_33175	48	ApGST-Mu4^T^	3	1	3
		DN10786_c1_g2_i11_10292	46	ApGST-Mu6^T^	13	4	14.8

T GST Class predicted by phylogenetic analysis

H Class predicted by homology to isoforms.

In addition, 8 spots (1, 2 and 6–11), corresponding to the GST 2DE profile, demonstrated strong reactivity to anti-Mu class antibody (Fig. 4). This reactivity was consistent across the 2 biological replicates. The 3 remaining spots (3–5) identified on the 2DE profiles did not demonstrate clear reactivity to the Mu class antibody. However, spots 3–5 were identified as Mu class GSTs *via* mass spectrometry with all 3 spots containing ApGST-Mu3. The classification of Mu class GST representatives from 2D analysis is again consistent with the dominance of Mu class representatives observed in the transcriptome and localized in the *A. perfoliata* whole cytosolic proteome.

No visible reaction from 2DE replicates probed with the anti-FhGST-S1 antibodies was observed (Fig. 4). The lack of reaction is consistent with the absence of Sigma class GSTs identified amongst the GSH agarose purified cytosolic proteins *via* LC-MSMS. Thus, confirming that Sigma GSTs were not purified from the cytosolic protein using GSH affinity.

## Discussion

GSTs are multifunctional enzymes and binding proteins that play key roles as the major detoxification system of internally derived toxins and xenobiotics in parasitic helminths (Brophy *et al*., 1989*a*; Brophy and Barrett, 1990*b*), likely as a consequence of limited Phase 1 oxygen dependent cytochrome P450 activities (Nelson, 2009; Pakharukova *et al*., 2012; Tsai *et al*., 2013). Until recently, our knowledge of the GST complement within *A. perfoliata* has been limited with GSTs only demonstrated in the ESP fractions (Wititkornkul *et al*., 2021; Hautala *et al*., 2022). Therefore, high-resolution sub-proteomics, coupled with bioinformatics, were utilized to further expand our knowledge, and understanding of the GST soluble superfamily within this equine helminth. Bioinformatic analysis has additionally identified 3 GST classes within the recent *A. perfoliata* transcriptome including those homologous with recognized Sigma, Omega and Mu class GSTs whilst members representing the Zeta, Delta, Epsilon, Theta, Alpha, Kappa and Pi GST classes were not identified. Mining the high-resolution proteome data of Wititkornkul *et al*. (2021) provided additional evidence for Mu and Omega class GSTs in the *A. perfoliata* secretome with, for the first time, 3 characterized GST classes identified within the *A. perfoliata* cytosolic proteome.

An assessment of the *A. perfoliata* transcriptome revealed 83 putative GSTs were classified as belonging to the Mu class GSTs making them by far the most abundant class present; with all representatives clustering well in the Cestode Mu class GST group. In support of the current analysis, a strong relationship of cestode GSTs classified as Mu class has been previously noted amongst alternative cestode species (Brophy *et al*., 1989*a*; Brophy and Barrett, 1990*b*) and an expansion of this GST class has been identified in the genomes of the cestodes *E. multilocularis*, *E. granulosus*, *T. solium* and *H. microstoma* (Tsai *et al*., 2013). Similarly, in the liver fluke *F. gigantica* Mu class GSTs were also identified as the most abundant (Morphew *et al*., 2012). The large proportion of *A. perfoliata* Mu class GSTs in comparison to the other classes accounts for their dominance localized across the whole worm proteome, GSH-affinity purified fractions, and the mined secretome. However, it can be noted that differences were observed between the unique Mu class ApGST sequences identified in the whole cytosolic proteome and the GSH affinity purified GSTs. These differences may likely be explained by the different enrichment in GSTs between the biological samples used. In addition, some Mu class GST, such as FhGST-Mu5 in *F. hepatica*, have been shown to be a ‘low-affinity’ GST isoform which presents a failure to purify though affinity columns (Stuart *et al*., 2021). Thus, the *A. perfoliata* ApGST-ome likely contains ‘low-affinity’ GST isoforms such as ApGST-Mu 5.

The 3 Sigma class GSTs within the transcriptome were previously identified by Wititkornkul *et al*. (2021) with Wititkornkul and colleagues defining ApGST-S1 as a likely Sigma class GST. ApGST-S1 was also identified in the cytosolic proteome. However, a lack of Sigma class GSTs was observed in the GSH-affinity purified fraction through both MS/MS and immunoblotting with Anti-FhGST-S1. This is likely due to a combination of a single Sigma identified within the cytosol in relatively low abundance and the dominance of Mu class GST present. In addition, only a single purification method was utilized, namely GSH-agarose affinity chromatography. Studies have demonstrated that *F. hepatica* Sigma GSTs have reduced binding to GSH-agarose affinity if large quantities of Mu class GSTs are present (Chemale *et al*., 2006; Morphew *et al*., 2012) and the combination use of GSH-agarose and S-hexyl GSH-agarose affinity chromatography can provide a more complete profile of helminth GSTs (Chemale *et al*., 2006; Morphew *et al*., 2012). Utilizing this combination method, a novel Sigma GST was identified in *F. gigantica* (Morphew *et al*., 2012). Thus, the use of GSH-affinity purifications may partly account for the lack of Sigma class GSTs identified post purification and further investigations may benefit from the incorporation of S-hexyl GSH-agarose affinity purification.

To date, limited research has focused on how *A. perfoliata* interacts with its host and the surrounding environment with only a recent elucidation toward the ability of *A. perfoliata* to modulate their host immune system (Lawson *et al*., 2019). ApGST localization demonstrated Mu, Sigma and Omega ApGSTs across the somatic (ApGST-Mu2-9, Mu11-12, Mu15, Mu17, Mu19, ApGST-O3-4 and ApGST-S1) and secreted (EVs: ApGST-Mu3, ESP: ApGST-Mu1, Mu3, Mu11, ApGSTO4 and ApGST-S1) proteomes, with ApGST-Mu3 showing to be the most dominant Mu GST identified across all proteome fractions and 2DE spots. Thus, this localization provides an insight into an important detoxification component for *A. perfoliata* survival as well as immunomodulators. Interestingly, Hautala *et al*. (2022) also identified GSTs in *A. perfoliata* ESP albeit searching against the NCBInr database and thus relying on homology and protein conservation. Utilizing BLAST demonstrates the closest protein match identified showed significant homology with ApGST-15 sequences were identified. Here, ApGST-15 was identified in only the somatic proteome fractions. Differences here again may be explained by difference in GST enrichment in GSTs between samples.

*A. perfoliata* Omega class GSTs were analysed separately based on the multiple sequence alignment, phylogenetics analysis and secondary characteristic structure prediction. In total, the analysis of the *A. perfoliata* transcriptome data revealed the presence of 8 novel *A. perfoliata* GST sequences, corresponding to 7 Omega class members, all of which showed strong homology to recognized platyhelminth Omega GSTs and Stringent starvation protein A (SspA). Of the 7 novel *A. perfoliata* Omega class GSTs, 2 (ApGST-O3 & ApGST-O4) and 1 (ApGST-O4) were localized within the whole cytosolic and EV-depleted ESP proteomes, respectively. Whilst Omega GSTs have been identified in several helminth species (Girardini *et al*., 2002; Morphew *et al*., 2012; Kim *et al*., 2016), previous studies have suggested they were likely absent from cestodes (Iriarte *et al*., 2012; Lopez-Gonzalez *et al*., 2018). However, proteins labelled SspA, an Omega GST homologue, have also been identified in cestodes species (Kim *et al*., 2016). Originally identified in bacteria, true SspA's present the characteristic GST fold but lacks GST binding activity due to replacement of the catalytic cystine residue (Hansen *et al*., 2005).

The secondary characteristic structure prediction established the consistency and similarity of the *β*-strand, *α*-helix and random coils structures within the recognized platyhelminth Omega class GST sequences, which are conserved regions of these proteins. Although all 8 novel *A. perfoliata* Omega class GSTs lacked the proline-rich residues in the Omega class characteristic N-terminal extension (PXXP motif) (Morphew *et al*., 2012). However, each putative Omega representative contained the catalytic cysteine residue characteristic of Omega class GSTs at position 32 (Cys32) (Morphew *et al*., 2012; Meng *et al*., 2014; Kim *et al*., 2016) and contained amino acid residues related to the Omega class GST signature motifs identified by Chemale *et al*. (2006). In addition, structural alignment analysis in Kim *et al*. (2016) and the ApGSTs demonstrates these catalytic residues and motifs were also identifiable in the designated SspA's from the cestodes *H. microstoma*, *E. granulosus* and *E. multilocularis*. Thus, this provides further support that cestode SspA's, in addition to the *A. perfoliata* sequences, belong to the Omega class of GSTs. Helminth Omega class GSTs have been demonstrated to have critical roles in protection against oxidative stress, the helminth reproduction system, and inhibition of macrophage viability (Meng *et al*., 2014; Kim *et al*., 2016; Wang *et al*., 2022). As with Mu and Sigma class GSTs, the presence of Omega class GSTs in the somatic and EV-depleted ESP fractions suggests they could play a vital role in *A. perfoliata*'s reproductive health and potential immune modulation function.

Previously, Zeta class GSTs have been identified in platyhelminthes such as *F. hepatica* and *F. gigantica*, the role of which is poorly understood (Morphew *et al*., 2012; Stuart *et al*., 2021). Zeta class GSTs do not appear to have been identified in cestode species and in agreement. Bioinformatic analysis of the *A. perfoliata* transcriptome did not return any GSTs related to the Zeta class. A preliminary homology search using BLAST across the Wormbase Parasite databases of Zeta GSTs against cestode species also retrieved no hits. Thus, it could be suggested that Zeta GSTs are likely absent from cestode species.

The high GSH affinity ApGST fraction purified through GSH agarose accounted for approximately 0.33% of the overall cytosolic proteins. In contrast, cestode and trematode GST have been shown to account for up to 3–4% of the soluble cytosolic protein fractions (Brophy *et al*., 1989*a*, 1989*b*; Vibanco-Pérez *et al*., 1999; Chemale *et al*., 2006; Duncan *et al*., 2018). However, a significant proportion of activity was left unpurified. Neither Sigma nor Omega class ApGSTs were purified through the GSH-affinity columns which will have contributed to GST activity. Additionally, previous studies have identified a failure of pure GST from pure cytosol extracts to completely bind to GSH-agarose affinity chromatography (Brophy and Barrett, 1990*a*; Stuart *et al*., 2021). Future investigation with improved GST purification, such as the addition of alternative chromatography methods, is thus likely warranted. ApGSTs level of specific GST activity in the CDNB assays was comparable to *H. diminuta* GST activity post affinity chromatography (Brophy and Barrett, 1990*a*). However, the GST activity was much higher than previously demonstrated among other cestodes and trematode species (Brophy *et al*., 1989*a*; Brophy and Barrett, 1990*a*; Duncan *et al*., 2018). In addition, ApGST demonstrated relatively high activity towards the model substrate EA to that previously shown in Sm28GST and recombinant FhGST (Walker *et al*., 1993; LaCourse *et al*., 2012). EA activity is traditionally used as a marker for mammalian Pi class GST, a common GST class expressed in the mammalian intestinal system (Mannervik *et al*., 1985). However, no Pi GST representatives were identified in transcriptome yet *A. perfoliata* GSTs, potentially Mu class, may exhibit similar detoxifying activity, potential evidence of a GST adapting to the mammalian xenobiotic environment.

In conclusion the data obtained from the *A. perfoliata* transcriptome and bioinformatics analysis demonstrate strong evidence that the *A. perfoliata* GST superfamily comprises of Mu, Sigma and Omega class representatives. In addition, proteomic analysis has confirmed the expression of ApGSTs across the *A. perfoliata* cytosolic and secretome proteomes. The discovery of all 3 cytosolic GST classes in the current data provides a greater in-depth characterization of the Equine tapeworm *A. perfoliata*, specifically the *A. perfoliata* GST-ome, illuminating potential mechanisms involving the parasites survival and host–parasite relationships. Given the importance of GSTs to helminth survival, the characterization of the *A. perfoliata* GST-ome provides valuable targets for developing further novel anthelminthics as well as potential usage in vaccine development for this often-neglected Equine tapeworm.

## Supporting information

Northcote et al. supplementary material 1 Northcote et al. supplementary material

Northcote et al. supplementary material 2 Northcote et al. supplementary material

Northcote et al. supplementary material 3 Northcote et al. supplementary material

Northcote et al. supplementary material 4 Northcote et al. supplementary material

Northcote et al. supplementary material 5 Northcote et al. supplementary material

Northcote et al. supplementary material 6 Northcote et al. supplementary material

## Data Availability

The *A. perfoliata* somatic proteome mass spectrometry proteomics data have been deposited to the ProteomeXchange Consortium (http://www.ebi.ac.uk/pride/archive/) *via* the PRIDE partner repository with the data set identifier PXD029998 and 10.6019/PXD029998. The *A. perfoliata* transcriptome can be provided upon reasonable request or alternatively is available at https://sequenceserver.ibers.aber.ac.uk/ for interrogation.

## Supplementary material

The supplementary material for this article can be found at https://doi.org/10.1017/S0031182024000015.
