# Supplementary material for: A dominance of Mu class glutathione transferases within the equine tapeworm Anoplocephala perfoliata
Source: Parasitology. 2024 Jan 11;151(3):282–94. doi: 10.1017/S0031182024000015 (PMC11007280; doi:10.1017/S0031182024000015)
Supplement: Northcote et al. supplementary material 1 — Northcote et al. supplementary material [file S0031182024000015sup001.pdf]

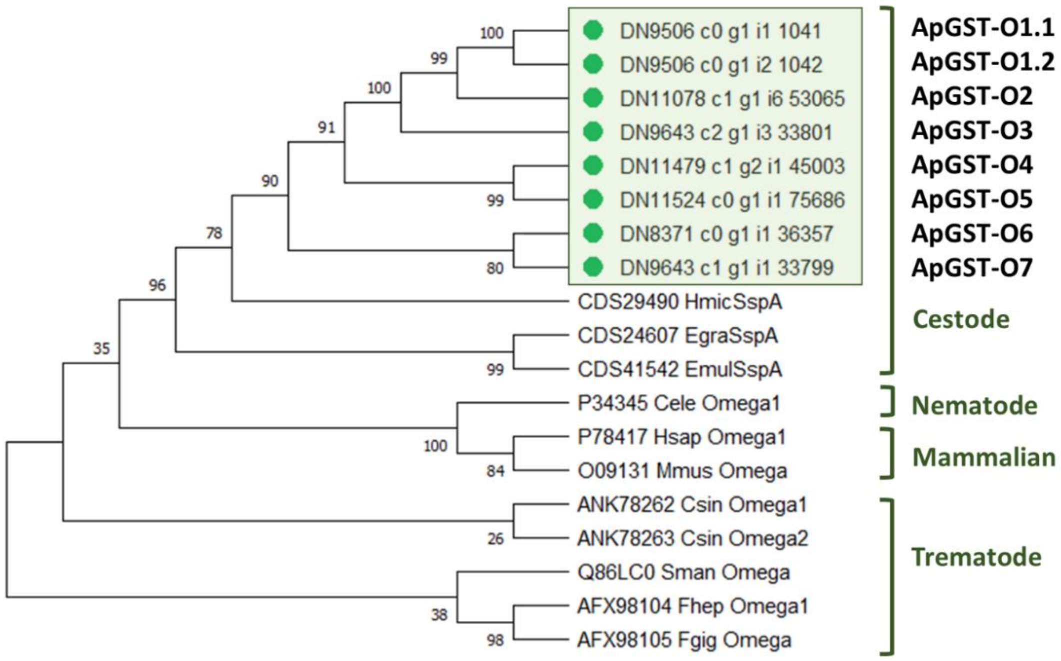

Supplementary Figure 1. Maximum likelihood (ML) tree with JTT matrix-based model inferred from *A. perfoliata* Omega class GST amino acid sequences. The bootstrap consensus tree inferred from 1000 replicates. Initial tree(s) for the heuristic search were obtained automatically by applying Neighbor-Join and BioNJ algorithms to a matrix of pairwise distances estimated using a JTT model, and then selecting the topology with superior log likelihood value. A discrete Gamma distribution was used to model evolutionary rate differences among sites (5 categories (+G, parameter = 1.9774)). This analysis involved 19 amino acid sequences. There was a total of 279 positions in the final dataset. Evolutionary analyses were conducted in MEGA X.
