# Supplementary material for: A dominance of Mu class glutathione transferases within the equine tapeworm Anoplocephala perfoliata
Source: Parasitology. 2024 Jan 11;151(3):282–94. doi: 10.1017/S0031182024000015 (PMC11007280; doi:10.1017/S0031182024000015)
Supplement: Northcote et al. supplementary material 2 — Northcote et al. supplementary material [file S0031182024000015sup002.pdf]

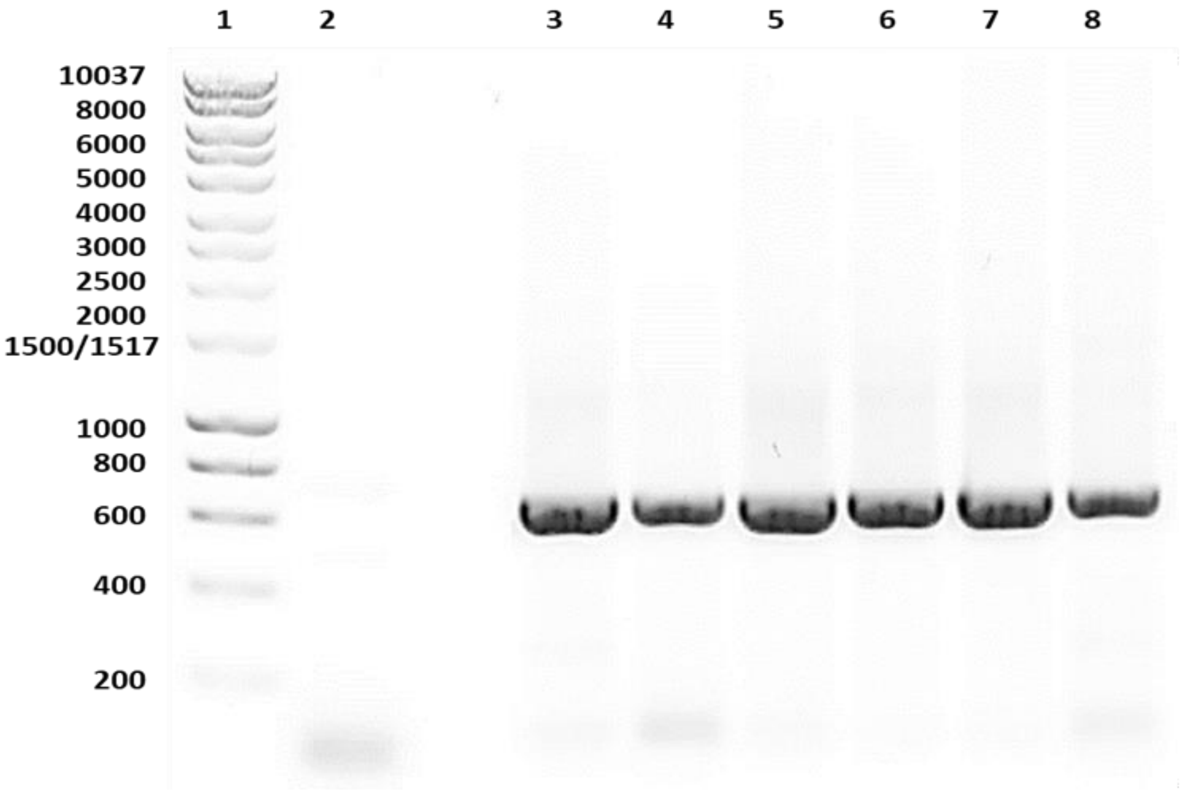

Supplementary Figure S2. Confirmation of *A. perfoliata* as the target equine tapeworm prior to GST purification. The ITS2 region was amplified as previously described (REF) and the PCR product run on 1% w/v agarose gel electrophoresis. (1) 1 kb DNA Ladder (Bioline) (2) Negative control, (3-8) Bands representing the amplified PCR product from 6 replicate tapeworms. All bands were cut for direct inhouse sequencing and species confirmation.
